# Diagnosis of Muscle Fatigue Using Surface Electromyography and Analysis of Associated Factors in Type 2 Diabetic Patients with Neuropathy: A Preliminary Study

**DOI:** 10.3390/ijerph18189635

**Published:** 2021-09-13

**Authors:** So Young Park, Chan Hyuk Park

**Affiliations:** 1 Department of Endocrinology and Metabolism, Kyung Hee University Hospital, Seoul 02447, Korea; malcoy@hanmail.net; 2 Department of Physical & Rehabilitation Medicine, Inha University Hospital, Incheon 22332, Korea

**Keywords:** type 2 diabetes mellitus, diabetic neuropathy, surface electromyography, fatigue

## Abstract

Diabetic neuropathy (DN) is a major complication associated with diabetes mellitus (DM) and results in fatigue. We investigated whether type 2 diabetic patients with or without neuropathy experienced muscle fatigue and determined the most influencing factor on muscle fatigue. Overall, 15 out of 25 patients with type 2 DM were diagnosed with DN using a nerve conduction study in the upper and lower extremities, and the composite score (CS) was calculated. We obtained the duration of DM and body mass index (BMI) from subjects, and they underwent a series of laboratory tests including HbA1c, fasting plasma glucose, triglycerides, and high- and low-density lipoprotein. To qualify muscle fatigue, this study used surface electromyography (sEMG). Anode and cathode electrodes were attached to the medial gastrocnemius. After 100% isometric maximal voluntary contracture of plantarflexion, the root mean square, median frequency (MDF), and mean power frequency (MNF) were obtained. We showed a correlation among laboratory results, duration of DM, BMI, CS, and parameters of muscle fatigue. The duration of DM was related to fatigue of the muscle and CS (*p* < 0.05). However, CS was not related to fatigue. The MDF and MNF of muscle parameters were positively correlated with HbA1c and fasting plasma glucose (*p* < 0.05). In conclusion, we suggest that the duration of DM and glycemic control play important roles in muscle fatigue in patients with DN. Additionally, sEMG is useful for diagnosing muscle fatigue in patients with DN.

## 1. Introduction

Diabetic neuropathy (DN) is a peripheral nerve dysfunction, and one of the major complications associated with diabetic retinopathy and diabetic nephropathy [1,2]. DN has a high prevalence among diabetic patients [2,3]. In particular, this is more common in patients with type 2 diabetes mellitus (DM) [2]. DN induces symmetrical neuropathic pain which is manifested as a stocking and glove pattern and presents with fatigue and sensory disturbances with motor disturbances being seen in more severe conditions inducing distal weakness and muscle atrophy of the lower legs and feet [2,4,5]. Fatigue is a common complication and leads to an energy imbalance [6,7], in addition to affecting the quality of life and functional status in people with type 2 DM [8,9].

Muscle fatigue is considered a multifaceted phenomenon consisting of physical and chemical changes in muscles which are distinct from alterations in the nervous system efficiency underlying the symptoms, causes, and mechanisms. Muscle fatigue is defined as a decreased maximum capacity to produce force or power output [10]. A range of methods has been used to analyze muscle fatigue, including muscle biopsy, muscle imaging, exercise endurance tests, and isometric strength tests. Due to its non-invasiveness nature, real-time data, and applicability, surface electromyography (sEMG) is a widely used technique to assess muscle fatigue [11,12]. The root mean square (RMS), mean power frequency (MNF), and median frequency (MDF) obtained from sEMG are useful parameters for assessing muscle fatigue [10,13,14].

A previous study showed that weakness of the ankle plantar and dorsal flexors was progressive and contributed to the severity of neuropathy in patients with symptomatic diabetic neuropathy [4]. The aim of this study was to study the correlation among various parameters, including laboratory studies, duration of diabetes mellitus (DM), body mass index (BMI), nerve conduction studies (NCS), and muscle fatigue (RMS, MDF, and MNF) diagnosed using sEMG in type 2 diabetic patients with and without neuropathy, as well as to investigate the relationship between DN and muscle fatigue and identify its most influencing factors.

## 2. Materials and Methods

### 2.1. Participants

Twenty-five patients with type 2 DM who were not previously diagnosed with DN were enrolled in this study. Patients with liver disease, renal disease, chronic alcoholism, and a history of chemotherapy or spine surgery were excluded. Patients with suspected or diagnosed psychological factors related to fatigue [15], spine disease, and vascular, neurological, or metabolic conditions unrelated to DM or DN were also excluded This study was performed retrospectively and in accordance with the Declaration of Helsinki; the study protocol was approved by the Inha University Hospital Institutional Review Board (approval number: 2019-10-018) on 18 November 2019.

Patients with type 2 DM were divided into two groups: with or without DN. Before NCS, all subjects had their history taken, including the duration of DM, and their BMI was assessed. Laboratory tests including HbA1c, fasting plasma glucose (FPG), albumin, creatinine, triglyceride (TG), high-density lipoprotein (HDL), and low-density lipoprotein (LDL), were performed. DN examination was performed using NCS. The method of NCS was the same as that reported in a previous study [1,6], using Keypoint electromyography (Dantec, Skovlunde, Denmark), and the temperature of patients during NCS was maintained above 32 °C. This study performed NCS in the upper and lower extremities with more severe sensory or motor symptoms. Examination of motor nerve function used the median, ulnar, posterior tibia, and peroneal nerve. The onset latency, amplitude, velocity, and minimal F-M latency were measured in these nerves. The peak latency and peak-to-peak amplitude were determined from a sensory examination on the median, ulnar, superficial peroneal, and sural nerves. A diagnosis of DN was determined on the basis of abnormal findings, in which a difference of more than two standard deviations above the normal value (Table 1) was observed using the method reported by Dick et al. [16]. DN was said to occur after identifying three or more abnormal findings among the onset latency, amplitude, conduction velocity, and F-latency in more than two of the median, ulnar, peroneal, sural, and tibia nerves. The composite score (CS) has previously been used to quantify NCS [1,6,17]. In previous studies, CS was defined as an objective measure by quantifying the degree of damage to the peripheral nerves in diabetic neuropathy [1]. As in the previous study, we calculated CS using the onset latency, amplitude, and velocity of the peroneal nerve, the distal latency of the tibia nerve and the amplitude of the sural nerve, comparing the results with the normal values of the hospital (Table 1). Additionally, we used the following scores: 0 below the 95th percentile, 1 from the 95th to 99th percentile, 2 from the 99th to 99.9th percentile, and 3 above the 99.9th percentile; all scores were added together and divided by 5. An increase in CS indicates severe neuropathy [1].

### 2.2. Exercise Protocol

**Figure 1:**
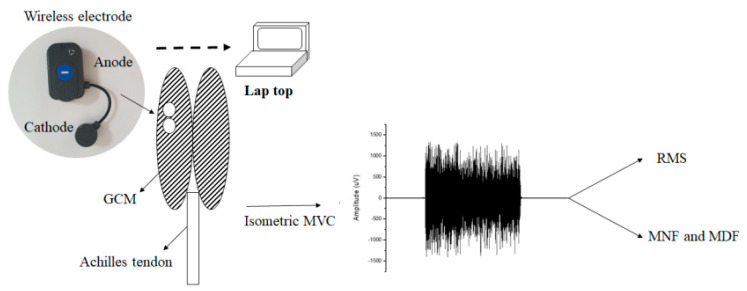
Surface electromyography detection and analysis. This schematic illustration shows the positioning of surface electrodes using wireless electrode devices. Signals obtained during isometric MVC. Note: GCM, medial gastrocnemius; MVC, maximal voluntary contraction; RMS, root mean square; MNF, mean power frequency; MDF = median frequency.

Participants were placed in the prone position. We used the gastrocnemius medial (GCM) to evaluate muscle fatigue from a previous study [18]. The skin of the subjects was cleaned with alcohol wipes before fixation of the electrodes [10]. The Ag/AgCl surface electrodes (diameter: 30 mm) were placed with the anode attached to the motor point of GCM, and the cathode 2 cm from the anode (Figure 1). To evaluate muscle fatigue, the subjects performed maximal ramp contraction (ramp-up rate: 5% MVC/sec) after a resting time of 10 min. When the isometric maximal voluntary contraction (MVC) was 100%, the participant was asked to sustain it for 30 s. To obtain the MVC of the subjects, they performed maximal plantarflexion for a set time.

### 2.3. Signal Processing

The RMS, MDF, and MNF, by plantarflexion in the prone position, were measured using the sEMG system (EMGworks 4.0 Analysis program, Delsys, Germany). The removal of noise generated by the electrocardiogram was performed using 50–500 Hz band-pass filtering, and sampling was set to 1,000 Hz. Using filtering techniques, artifacts were eliminated. To investigate fatigue, the RMS (unit: µV), MDF (unit: Hz) and MNF (unit: Hz) were calculated after the “Fourier transform” and were analyzed using filtered data. Distributions of RMS explain the action potential energies during the contractions, defined as below [10]:

$$\mathrm{RMS}=\sqrt{\frac{\sum_{ί=1}^{n}│{\mathrm{rawData}}_{ί}│}{n}}$$

where ί is the procedure number of the processing sample, rawDdata_ί_ is the value of the ί-th sample point, and n is the total number of data points).

The most representative valuable frequency-domain characteristics are MNF and MDF. For the evaluation of muscle fatigue in sEMG signals, MDF and MNF are defined as follows [10]:

$$\int_{0}^{\mathrm{MDF}}P\left(t,\omega \right)d\omega =\int_{\mathrm{MDF}}^{\infty }P\left(t,\omega \right)d\omega \;=\frac{1}{2}\int_{\mathrm{MDF}}^{\infty }P\left(t,\omega \right)d\omega \;\mathrm{MNF}=\frac{\int_{\;0}^{\infty }\omega P\left(t,\omega \right)d\omega }{\int_{0}^{\infty }P\left(t,\omega \right)d\omega }$$

where (t,ω) is the power spectrum of EMG signals based on wavelet packet transformation.

### 2.4. Statistical Analysis

Quantitative data are reported as the mean ± standard deviations, and statistical analysis was performed using the SPSS software (version 26.0; SPSS, Chicago, IL, USA). The baseline characteristics in patients with and without DN were compared using an independent sample *t*-test. To identify a normal distribution, the Kolmogorov–Smirnov test was used on all data. Pearson correlation analyses were also conducted. A *p*-value of <0.05 was considered statistically significant.

## 3. Results

Among the 25 subjects with DM, 15 subjects were diagnosed with DN. The comparison between subjects with DN and subjects without DN was investigated using a *t*-test after verification of a normal distribution using the Kolmogorov–Smirnov test. Subjects with DN had lower albumin levels (Table 2, *p* < 0.05). Although the mean HbA1c and FPG were higher in patients with DN than in patients without DN, the difference was not statistically significant (*p* > 0.05).

CS, which indicates the severity of DN, was higher in the subjects with DN (mean ± SD: 0.70 ± 0.45) than those without DN (mean ± SD: 0.07 ± 0.10, *p* < 0.05). The mean MDF (*p* = 0.028) and MNF (*p* = 0.027) in subjects with DN were significantly lower than in the subjects without DN. However, the RMS between groups was not statistically significant (*p* > 0.05, Table 3).

The relationship between various parameters in subjects with DN was investigated using Pearson’s correlation coefficients after verification of a normal distribution using the Kolmogorov–Smirnov test. CS was correlated with duration (*r* = 0.596, *p* < 0.05) and serum creatinine level (*r* = 0.601, *p* < 0.05) but did not show a significant difference from fatigue parameters obtained using sEMG (Table 4, MDF: *r* = −0.354, MNF: *r* = −0.298, *p* > 0.05). The MDF and MNF were correlated with FPG, HbA1c, and duration. However, RMS was not associated with these measurements. The correlation coefficient between parameters of muscle fatigue (MDF: *r* = −0.794, MNF: *r* = −0.813, *p* < 0.05) and duration was higher than between indicators of muscle fatigue and glycemic control (Table 4, *p* < 0.05).

## 4. Discussion

This study found that in patients with DN there was a high likelihood of muscle fatigue, and the duration of DM and glycemic control were responsible for muscle fatigue. However, there was no correlation between the severity of neuropathy and muscle fatigue. Additionally, the serum albumin level between patients with and without DN was significantly different. This is consistent with a previous study that indicated that albumin changes due to oxidative stress were a representative biomarker for DN [19].

This study showed that CS in patients with DN was related to the duration of DM and serum creatinine level. Several studies have explained the high prevalence of neuropathy in patients as being dependent on age, glucose control, and duration of diabetes [4,20]. The presence of peripheral neuropathy increased with serum creatinine [21]. DN is induced by the progressive degeneration of peripheral nerve axons [22]. The pathophysiology of DN involves increased glucose and lipids which induce vascular dysfunction, causing a decrease in nerve blood flow and an increase in endoneurial hypoxia [23,24]. Our previous study also showed that CS was positively related to the duration of disease, and this indicates that the severity of neuropathy (CS) increases with the increasing duration of DM [1]. However, the evidence that hyperglycemia causes axonal atrophy which leads to DN is inconsistent with our findings, which showed no significant correlation between CS and HbA1c or FPG [25]. This might be because of the small scale of our study; as such, further evaluation is necessary. Therefore, our findings are consistent with previous evidence suggesting that the duration of DM is responsible for DN.

This study quantified muscle fatigue using sEMG. MDF and MNF showed a significant decrease, but RMS was not significantly different between patients with and without DN. Muscle fatigue is a subjective symptom, which is defined as a decrease in objective performance, as well as a decreased maximum capacity to provoke force or power output, as measured by sEMG [5,10]. Muscle fatigue occurred more rapidly in the absence of glucose. We postulate that the association between low muscle glycogen and impaired contractile function indicates that glycogen is a necessary substrate, the depletion of which causes a decrease in the rate of ATP regeneration [26]. The association between hypoglycemia and fatigue has previously been described in applied physiology studies [14,27]. Another study showed that acute hypoglycemia is related to higher levels of fatigue [5,28]. The present results are consistent with this evidence since MDF and MNF showed a positive correlation with FPG and HbA1c in patients with DN. However, because RMS indicated a low fatigue sensitivity, the RMS was not significantly different [10]. In addition, there were no significant differences between CS and the parameters of muscle fatigue (RMS, MDF, and MNF). We hypothesized that small fibers could not be detected, as our previous study demonstrated a correlation between fatigue and nerve fiber function since NCS detects large nerve fibers. Small nerve fibers or minor defects in peripheral nerves contribute to the pathophysiological mechanism of fatigue in patients with DN [29]. Thus, to illustrate this, further studies are required. In addition, a previous study reported that because MNF is always higher than MDF due to the skewed shape of the EMG power spectrum, MDF estimation is more affected by muscle fatigue than by random noise, particularly noise located in the high-frequency band of the EMG power spectrum [13]. Additionally, in MNF, there are relatively more type 1 muscle fibers, whereas RMS is positively correlated with type 2 muscle fibers [30]. Our findings are consistent with this previous result and suggest that muscle fatigue is influenced by type 1 muscle fibers.

To the best of our knowledge, this is the first study to demonstrate that muscle fatigue, NCS, and parameters of DM are correlated in patients with DN. DN was associated with the duration of DM. Moreover, muscle fatigue and CS in DN patients were greater in those with a longer duration of DM, but CS was not directly related to muscle fatigue.

There were some limitations in this study. Firstly, this study was a preliminary study, and a large-scale study is required to confirm the results. Secondly, as muscle atrophy in patients with DM contributes to protein degradation in the muscle and induces fatigue, a further study of the relationship between fatigue and muscle atrophy is required [7]. Thirdly, muscle fatigue does not represent general fatigue; therefore, a further study of the relationship between isolated muscle fatigue and performance tests (e.g., six-minute walk test) is required [31]. Fourthly, because this study compared and analyzed values between type 2 diabetic patients with and without DN, there was no normalization as a function of the maximal contraction in the movement studied or as a function of the MVC. Fifthly, this study did not consider the effect of BMI. Lastly, despite removing signal artifacts, there remains a question of whether they were completely eliminated. Thus, further evaluation is required.

## 5. Conclusions

We investigated whether type 2 diabetic patients with or without neuropathy experienced muscle fatigue and determined the most influencing factor on muscle fatigue. In conclusion, we suggest that fatigue in patients with DN is related to the duration of DM and glucose control, and that sEMG is a useful tool to diagnose fatigue in patients with DN.

## Figures and Tables

**Table 1:** Electrophysiological criteria for abnormal nerve conduction study.

**Median Motor Nerve**	**Median Sensory Nerve**	**Ulnar Motor Nerve**	**Ulnar Sensory Nerve**
L > 4.0 ms	L > 3.5 ms	L > 3.8 ms	L > 3.4 ms
A < 5.0 mV	A < 10.0 μV	A < 5.0 mV	A < 7.5 μV
CV < 49.0 m/s		CV < 49.0 m/s	
MF > 24.2 ms		MF > 24.8 ms	
**Peroneal Motor Nerve**	**Superficial** **Peroneal Sensory Nerve**	**Tibial Motor Nerve**	**Sural Sensory Nerve**
L > 4.5 ms	L > 3.5 ms	L > 5.0 ms	L > 3.5 ms
A < 1.0 mV	A < 3.7 μV	A < 5.0 mV	A < 5.0 μV
CV < 40.0 m/s		CV < 40.0 m/s	
MF > 45.0 ms		MF > 45.3 ms	

Note: L, latency; A, amplitude; CV, conduction velocity; MF, minimal F-M latency.

**Table 2:** Clinical characteristics of DM patients with or without DN. (* *p* < 0.05).

Characteristic	DM − DN (*n* = 10)	DM + DN (*n* = 15)	*p*-Value
Sex, men/women	5/5	8/7	
Age (years)	45.67 ± 11.44	48.00 ± 16.24	0.615
Duration of DM, years	5.23 ± 5.89	10.43 ± 6.47	0.066
Height (cm)	165.63 ±11.24	166.16 ± 8.20	0.897
Weight, kg	76.09 ± 18.88	68.99 ± 19.18	0.394
BMI (kg/m^2)^	27.62 ± 5.78	24.73 ± 5.33	0.233
Albumin (g/dL)	4.52 ± 0.35	4.01 ± 0.425	0.007 *
Creatinine (mg/dL)	0.96 ± 0.45	2.09 ± 3.35	0.328
HbA1c (%)	10.29 ± 2.48	10.35 ± 3.30	0.963
FPG (mg/dL)	192.33 ± 61.22	191.57 ± 91.71	0.983
TG (mg/dL)	180.56 ± 70.47	200.50 ± 170.85	0.744
HDL-cholesterol (mg/dL)	46.56 ± 8.23	45.71 ± 14.70	0.878
LDL-cholesterol (mg/dL)	106.00 ± 38.12	105.38 ± 39.71	0.968

Note: DM, diabetic mellitus; DN, diabetic neuropathy; BMI, body mass index; FPG, fasting plasma glucose; TG, triglyceride; HDL, high-density lipoprotein; LDL, low-density lipoprotein. Values are the mean ± standard deviation. * *p* < 0.05.

**Table 3:** NCS and muscle characteristics of DM patients with or without DN. (* *p* < 0.05).

Characteristic	DM − DN (*n* = 10)	DM + DN (*n* = 15)	*p*-Value
CS	0.07 ± 0.10	0.70 ± 0.45	0.000 *
RMS (μV)	191.79 ± 103.79	212.53 ± 86.23	0.608
MDF (Hz)	175.10 ± 37.38	131.99 ± 45.85	0.028 *
MNF (Hz)	190.88 ± 40.48	147.27 ± 44.13	0.027 *

Note: NCS, nerve conduction study; DM, diabetic mellitus; DN, diabetic neuropathy; CS, composite score; RMS, root mean square; MDF, median frequency; MNF, mean power frequency. Values are the mean ± standard deviation. * *p* < 0.05.

**Table 4:** Pearson’s correlation coefficient (*r*) between parameters and CS, RMS, MDF, and MNF using Pearson correlation analysis in patients with DN.

Components	Coefficient	CS	RMS (μV)	MDF (Hz)	MNF (Hz)
BMI	*r*	−0.334	0.248	−0.061	−0.086
Creatinine	*r*	0.601 *	0.172	−0.424	−0.418
Albumin	*r*	−0.447	0.264	−0.229	−0.204
FPG	*r*	0.134	0.308	0.581 *	0.628 *
TG	*r*	−0.204	−0.376	0.313	0.324
HDL	*r*	0.522	−0.015	−0.173	−0.136
LDL	*r*	0.224	0.239	0.009	0.089
HbA1c	*r*	0.224	0.118	0.668 *	0.672 *
Duration	*r*	0.596 *	0.133	−0.794 *	−0.813 *
CS	*r*	-	−0.265	−0.354	−0.298

Note: DN, diabetic neuropathy; CS, composite score; RMS, root mean square; MDF, median frequency; MNF, mean power frequency; FPG, fasting plasma glucose; TG, triglyceride; HDL, high-density lipoprotein; LDL, low-density lipoprotein; duration, the duration of DM. Values are the mean ± standard deviation. * *p* < 0.05.
